# Investigation of Possible Link between Interferon-α and Lyme Disease

**DOI:** 10.3201/eid2911.230839

**Published:** 2023-11

**Authors:** Armin Alaedini, Mary K. Crow, Brian A. Fallon, Elzbieta Jacek, Gary P. Wormser

**Affiliations:** Columbia University, New York, New York, USA (A. Alaedini, B.A. Fallon);; New York Medical College, Valhalla, New York, USA (A. Alaedini, G.P. Wormser);; Hospital for Special Surgery, New York (M.K. Crow);; The New York State Psychiatric Institute, New York (B.A. Fallon);; Lehigh Valley Health Network, Allentown, Pennsylvania, USA (E. Jacek)

**Keywords:** Interferon-α, Lyme disease, *Borrelia burgdorferi*, Post-treatment Lyme disease symptoms, bacteria

To the Editor: We were intrigued by Hernandez et al.’s recent important study linking persistent subjective symptoms after Lyme neuroborreliosis in Europe with increased interferon (IFN) α levels in blood (*1*). Their findings align with our earlier study in the United States, which showed an association between persistent objective neurocognitive deficits, despite antibiotic treatment for Lyme disease, and elevated blood IFN-α activity (*2*). In our study, we decided to evaluate the potential role of IFN-α in this particular group of patients with posttreatment Lyme disease symptoms (PTLDS), based on the extensive animal and human data that connect IFN-α with adverse cognitive, neuropsychiatric, and behavioral manifestations.

A noteworthy difference between the 2 studies is the method of IFN-α detection. Hernandez et al. (*1*) relied on a bead-based immunoassay to directly measure IFN-α levels, whereas we used a functional cell-based assay and quantitative real-time PCR to assess IFN-α activity (*2*,*3*). Direct quantitation of IFN-α can be challenging, as highlighted by the fact that concentrations for some study participants in the Hernandez et al. article appeared to fall below the limit of quantitation, thus complicating the analysis and interpretation of the data. However, newer ultrasensitive assays recently made available might be useful in detecting even very low concentrations of IFN-α in controls and in patients with PTLDS (*4*).

The observations linking PTLDS with increased IFN-α strengthen the growing evidence for the involvement of innate and adaptive immune-mediated pathways in post-Lyme disease sequela. Future studies can use newer ultrasensitive assays for IFN-α detection and be extended to include a more diverse population of persons with PTLDS, including those without a history of neuroborreliosis and those without cognitive dysfunction (*5*). The findings might have important implications for establishing biomarkers and even for potentially finding effective therapies for PTLDS, with possible relevance to other post-infection conditions.
